# Trastuzumab-deruxtecan and radiotherapy: a safety sub-analysis of COMBART prospective cohort study

**DOI:** 10.1093/oncolo/oyaf229

**Published:** 2025-07-24

**Authors:** Edy Ippolito, Martina Benincasa, Francesco Pantano, Carlo Greco, Marco Donato, Carla Maria Gullotta, Lucrezia Toppi, Rita Alaimo, Paola Martucci, Guenda Meffe, Michele Fiore, Giuseppe Tonini, Rolando Maria D’Angelillo, Sara Ramella

**Affiliations:** Research Unit of Radiation Oncology (Medicine and Surgery), Università Campus Bio-Medico di Roma, Rome, Italy; Radiation Oncology, Fondazione Policlinico Universitario Campus Bio-Medico, Rome, Italy; Research Unit of Radiation Oncology (Medicine and Surgery), Università Campus Bio-Medico di Roma, Rome, Italy; Research Unit of Medical Oncology (Medicine and Surgery), Università Campus Bio-Medico di Roma, Rome, Italy; Medical Oncology, Fondazione Policlinico Universitario Campus Bio-Medico di Roma, Rome, Italy; Research Unit of Radiation Oncology (Medicine and Surgery), Università Campus Bio-Medico di Roma, Rome, Italy; Radiation Oncology, Fondazione Policlinico Universitario Campus Bio-Medico, Rome, Italy; Medical Oncology, Fondazione Policlinico Universitario Campus Bio-Medico di Roma, Rome, Italy; Medical Oncology, Fondazione Policlinico Universitario Campus Bio-Medico di Roma, Rome, Italy; Radiation Oncology, Fondazione Policlinico Universitario Campus Bio-Medico, Rome, Italy; Radiation Oncology, Fondazione Policlinico Universitario Campus Bio-Medico, Rome, Italy; Radiation Oncology, Fondazione Policlinico Universitario Campus Bio-Medico, Rome, Italy; Radiation Oncology, Fondazione Policlinico Universitario Campus Bio-Medico, Rome, Italy; Research Unit of Radiation Oncology (Medicine and Surgery), Università Campus Bio-Medico di Roma, Rome, Italy; Radiation Oncology, Fondazione Policlinico Universitario Campus Bio-Medico, Rome, Italy; Research Unit of Medical Oncology (Medicine and Surgery), Università Campus Bio-Medico di Roma, Rome, Italy; Medical Oncology, Fondazione Policlinico Universitario Campus Bio-Medico di Roma, Rome, Italy; Radiation Oncology, Tor Vergata University, Rome, Italy; Research Unit of Radiation Oncology (Medicine and Surgery), Università Campus Bio-Medico di Roma, Rome, Italy; Radiation Oncology, Fondazione Policlinico Universitario Campus Bio-Medico, Rome, Italy

**Keywords:** Trastuzumab-Deruxtecan, radiotherapy, breast cancer, safety

## Abstract

**Aim:**

This observational analysis, derived from the prospective mono-institutional COMBART cohort (stage IV breast cancer patients undergoing radiation therapy during novel systemic treatments), evaluates the safety of combining radiotherapy (RT) with Trastuzumab Deruxtecan (T-DXd) in metastatic breast cancer patients.

**Material and methods:**

Patients eligible for this analysis received conventional RT or stereotactic radiotherapy (SRT) concurrently with T-DXd. RT was considered concurrent if administered on the same day as T-DXd or during the 3-week interval between cycles. T-DXd was given at a dose of 5.4 mg kg^−1^ via intravenous infusion every 3 weeks until progression or unacceptable toxicity. The primary endpoint was to assess RT-related acute and late toxicities, graded using the National Cancer Institute Common Terminology Criteria for Adverse Events (NCI CTCAE), version 5.0.

**Results:**

Forty patients who underwent RT or SRT concurrently with T-DXd were selected from the cohort of 145 patients enrolled in the COMBART trial. A total of 98 lesions were treated. Palliative RT was performed in 50.0% of patients, while 50.0% underwent SRT. Acute toxicity of any grade was observed in 8/40 patients (20.0%) during RT. One patient developed grade 3 anemia (3.3%), leading to RT discontinuation. Late toxicity occurred in 4/40 patients (10%) consisting of 3 radiation pneumonitis (RP) and 3 radionecrosis. Among the 22 patients treated with SBRT for oligoprogressive disease, the time from the initiation of RT to second disease progression (progression-free survival 2 -PFS2) was 11.3 months (95% CI, 4.61-25.82), and the median time to systemic treatment change was 19.1 months (95% CI, 12.7-25.56).

**Conclusions:**

The safety data for concurrent RT and T-DXd are promising. Most non-hematologic toxicities appear to be related to RT, while hematologic toxicities are likely influenced by T-DXd and should be closely monitored.

Implications for Practice The integration of Trastuzumab Deruxtecan (T-DXd) with radiotherapy (RT) in metastatic HER2-positive breast cancer demonstrates a favorable safety profile, with acceptable toxicity and promising progression-free survival outcomes for patients with oligoprogressive disease treated with SBRT. These findings suggest that concurrent T-DXd and RT could be a feasible therapeutic approach for selected patients, potentially extending the benefits of systemic treatment. Close monitoring for toxicity is recommended, especially in cases involving thoracic or brain irradiation.

## Introduction

Breast cancer is the most prevalent cancer among women and the leading cause of cancer-related death worldwide, with approximately 5%-10% of patients presenting with metastatic disease at diagnosis. Around 17% of breast tumors exhibit human epidermal growth factor receptor 2 (HER2) overexpression or amplification, which is often associated with an aggressive clinical phenotype.^1^

Over the last decade, HER2-directed antibody–drug conjugates (ADCs) have transformed the treatment landscape of both early-stage and advanced HER2-positive breast cancer. Trastuzumab Deruxtecan (T-DXd) is a HER2-targeted ADC comprising a potent topoisomerase I inhibitor (DXd) chemically linked to a humanized monoclonal antibody with the same amino acid sequence as trastuzumab.^2^^,^^3^

It has a high drug-to-antibody ratio (DAR) of approximately 8:1, as each antibody carries about 8 cytotoxic payload molecules; this, combined with a stable, tumor-selective linker, enables precise delivery of the cytotoxic payload to HER2-overexpressing tumor cells.^4^ Upon internalization, the payload is selectively cleaved by lysosomal enzymes, inducing DNA damage and apoptosis, with neighboring tumor cells also affected via a bystander effect.^4-6^

In the DESTINY-Breast03 trial, T-DXd demonstrated superior progression-free survival (PFS) compared to trastuzumab emtansine (T-DM1) in the second-line setting (28.8 months vs 6.8 months), making it the standard of care for metastatic HER2-positive breast cancer in this context.^7^ More recently, T-DXd has been approved for monotherapy in HER2-low ­metastatic breast cancer, showing significant improvements in both median PFS and overall survival when compared with physician-choice chemotherapy.^8^

T-DXd is associated with adverse events (AEs), including interstitial lung disease (ILD)/pneumonia, leukopenia, reduced left ventricular ejection fraction, polyserositis, increased transaminases, nausea, and alopecia.^3^^,^^9^ However, the safety and efficacy of combining T-DXd with radiotherapy (RT) remain unknown.

The DESTINY-Breast03 trial permitted palliative RT to metastatic sites, provided it did not interfere with treatment assessment or interruptions. Notably, thoracic irradiation was not allowed, and patients who received whole-brain radiotherapy were required to recover from acute RT effects before T-DXd initiation.^10^ Evidence on this combination is limited, with available data being primarily retrospective.^11-14^

Given the above considerations, this study aimed to prospectively evaluate the safety of concurrent RT and T-DXd in patients with metastatic breast cancer.

## Material and methods

### Study group

This study is an observational analysis based on the prospective mono-institutional COMBaRT trial. COMBaRT is an acronym for “Concomitant Radiotherapy and New Drugs in Metastatic Breast Cancer.” It is a prospective observational registry with all data collected prospectively. Patients enrolled are divided into 4 cohorts according to the drugs delivered concurrently: ADCs (cohort 1), anti-CDK4/6 (cohort 2), anti-HER2 (cohort 3), and immunotherapy (cohort 4). The primary aim of the study is to collect toxicity during RT combined with new drugs, while secondary outcomes are pain response after palliative RT treatments and PFS and response after SRT. In this analysis, the focus was to evaluate the safety of radiation treatment delivered concurrently with T-DXd. Radiation treatment was considered concurrent if delivered on the same day as T-DXd administration or during the 3-week interval between cycles. Eligible patients received either conventional RT or SRT and provided written informed consent to participate in the COMBART study. The study was approved by the Institutional Ethics Committee (Protocol PAR 73.23 OSS) and conducted in accordance with Good Clinical Practice (GCP), the International Council for Harmonisation GCP guidelines, and the ethical principles outlined in the Declaration of Helsinki and its amendments.

### Treatment

T-DXd was administered to patients with metastatic, hormone receptor (HR)-positive or HR-negative, HER2-positive breast cancer at a dose of 5.4 mg kg^−1^ via intravenous infusion every 3 weeks until progression or unacceptable toxicity. The primary endpoint of this study was to analyze radiation-related acute and late toxicities during and after RT in patients receiving concurrent systemic T-DXd therapy. All patients were treated with advanced techniques (SRT or VMAT). No 3D conformal radiotherapy was used, even for palliative treatment, as treatment planning prioritized dose conformity and organs at risk (OAR) sparing in a cohort receiving novel systemic therapies. Palliative treatment schedules included 30 Gy in 10 fractions, 20 Gy in 5 fractions, and 8 Gy in a single fraction. Patients with oligoprogressive-metastatic lesions were treated with SBRT delivered in 3-5 fractions.

### Statistics

Descriptive statistics were applied to summarize the data. Median values and ranges were reported for continuous variables, while counts and frequencies were used for categorical variables. Treatment-related AEs were categorized and graded using the National Cancer Institute Common Terminology Criteria for Adverse Events (NCI CTCAE), version 5.0. Dosimetric data for each patient were collected based on the primary treatment site and the type of radiation therapy (SBRT vs palliative). For patients treated for oligoprogression after the beginning of the T-DXd, progression-free survival 2 (PFS2) was defined as the time from the initiation of RT to second disease progression, and the median time to systemic treatment change was calculated from the initiation of RT to change of systemic treatment (T-DXd).

## Results

### Patient population

Forty patients who underwent RT or SRT concurrently with T-DXd were selected from the cohort of 145 patients enrolled in the COMBART trial. A total of 98 RT courses were delivered. All patients had HER2-positive tumors, and 56.1% were postmenopausal. T-DXd was used as a second-line treatment in 26.5% of patients, with the remaining receiving it as a subsequent line. Twenty-two patients (55.0%) were treated with ablative intent. See Table 1 for patient details.

**Table 1.** Patients and treatment characteristics.

	Patients, *n* (%)	RT treatment, *n* (%)	RT site	RT intent (Palliative/Not Palliative)	RT technique (SBRT, VMAT)
Total	40 (100)	98 (100)	Bone: 54 (55.1%) Brain: 21 (21.4%) Lung: 5 (5.1%) Other: 18 (18.4%)	Palliative: 49 (50.0%) Not palliative: 49 (50.0%)	SBRT: 52 (53.1%) VMAT: 46 (46.9gg%)
Menopausal status					
Premenopausal o Perimenopausal	17 (42.5)	43 (43.9)	Bone: 24 (55.8%) Brain: 13 (30.2%) Lung: 1 (2.3%) Other: 5 (11.6%)	Palliative: 18 (41.8%) Not palliative: 25 (58.2%) Palliative: 31 (56.3%)	SBRT: 25 (58.1%) VMAT: 18 (41.9%)
Postmenopausal	23 (57.5)	55(56.1)	Bone: 34 (61.8%) Brain: 8 (14.5%) Lung: 4 (7.3%) Other: 13 (23.6%)	Not palliative: 24 (43.7%)	SBRT: 27 (49.1%) VMAT: 28 (50.9%)
Line of therapy					
Second	16(40)	26 (26.5)	Bone: 13 (50.0%) Brain: 4 (15.4%) Lung: 3 (11.5%)	Palliative: 18 (69.2%) Not palliative: 8 (30.8%) Palliative: 31	SBRT: 8 (30.8%) VMAT: 18 (69.2%)
Others	24 (60)	72 (73.5)	Other: 6 (23.1%) Bone: 41 (56.9%) Brain: 17 (23.7%) Lung: 2 (2.7%) Other: 12 (16.7%)	(43.1%) Not palliative: 41 (56.9%)	SBRT: 44 (61.1%) VMAT: 28 (38.9%)
ECOG 0	28 (70)	59 (60.2)	Bone: 38 (64.4%) Brain: 8 (13.6%) Lung: 1 (1.7%) Other: 12 (20.3%)	Palliative: 35 (59.3%) Not palliative: 24 (40.7%)	SBRT: 25 (42.4%) VMAT : 34 (57.6%)
1	12 (30)	39 (48)	Bone: 16 (41.0%) Brain: 13 (33.3%) Lung : 4 (10.3%) Other: 6 (15.4%)	Palliative: 14 (35.9%) Not palliative: 25 (64.1%)	SBRT: 27 (69.2%) VMAT: ff12 (30.8%)
Mean duration (months) of TDxd therapy (median) to last follow-up	15.1 (range =2-39)				

Abbreviations: RT, radiotherapy; SBRT, stereotactic body radiotherapy; VMAT, volumetric modulated Arc therapy.

The median follow-up time was 11 months (range, 6-31 months). The median duration of T-DXd therapy was 16 months (range, 2-39 months). The median time from the initiation of T-DXd therapy to the radiation therapy course was 6.5 months (range, 0.5-27 months). Among 22 patients treated with SRT for oligoprogressive disease, occurring a median of 9.5 months (range, 4.6-26.8 months) after the initiation of T-DXd, the PFS2 was 11.3 months (95% CI, 4.61-25.82). In this group of patients, median time to systemic treatment change was 19.1 months (95% CI, 12.7-25.56).

### Radiotherapy treatments

Palliative RT was delivered to 50.0% of patients, while 50.0% received SBRT with ablative intent. Bone was the most frequently treated site. Dosimetric parameters based on treatment sites are given in Table 2.

**Table 2.** Treatment plan: dosimetric data.

	Mean	
Thoracic lesions	*N* = 34 (100%)	SD
PTV (cc)	122.9	271.9
**ChestWall**		
D_max_ 0.1 cc (Gy)	42.9	15.1
D_max_ 0.5 cc (Gy)	37.4	12.2
D30cc (Gy)	37.4	4.5
**Esophagus**		
D_mean_ (Gy)	2.0	2.6
D_max_ 0.1 cc (Gy)	9.4	7.2
D_max_ 0.5 cc (Gy)	8.9	7.0
**Heart**		
D_mean_ (Gy)	1.7	1.5
D_max_ 0.5 cc (Gy)	6.9	8.0
D15cc (Gy)	2.1	5.1
**Ipsilateral lung**		
D_mean_ (Gy)	3.2	4.2
**Contralateral lung**		
D_mean_ (Gy)	1.2	1.6
**Bilateral lungs**		
D_mean_ (Gy)	0.8	0.5
V20 Gy (%)	1.2	9.5
V5Gy (%)	4.8	3.8
**Spinal cord**		
D_max_ (Gy)	9.9	9.0
D 0.035 cc (Gy)	7.4	5.0
**Abdominal/pelvic lesions**	*N* = 43 (100%)	
PTV (cc)	157.9	263.0
**Stomach**		
D_max_ (Gy)	8.16	11.9
D_mean_ (Gy)	1.9	2.7
D_max_ 0.1 cc (Gy)	6.8	9.8
**Rectum**		
D_max_ 0.1 cc (Gy)	10.1	8.7
D 20 cc (Gy)	2.0	2.7
**Bladder**		
D_max_ 0.1 cc (Gy)	7.6	8.9
**Liver**		
D_mean_ (Gy)	10.3	8.4
D700cc (Gy)	4.5	5.0
V10 Gy (%)	58.63	21.8
**Kidneys**		
D_mean_ (Gy)	2.25	1.53
V20Gy (%)	0.0	0.0
vV10 Gy (%)	44.8	22.9
**Bowel**		
V15 Gy (cc)	4.7	5.2
V45 Gy (cc)	0.0	0.0
D_max_ (Gy)	14.9	10.9
**Spinal Cord**		
D_max_ (Gy)	3.0	3.1
D 0.035 cc (Gy)	2.3	3.4
**Cauda Equina**		
D0.035 cc (Gy)	13.1	12.0
**Brain lesions**	*N* = 21 (100%)	
PTV (cc)	38.2	237.2
**Brain**		
V14 Gy	20.4	18.1
D20cc (Gy)	12.2	6.3
V20 Gy (cc)	23.1	16.7
**Brainstem**		
D_max_ (Gy)	4.3	5.2
D0.035 cc (Gy)	2.71	2.18
**Optic Nerve**		
D0.035 (Gy)	1.91	2.62

Abbreviations: D_max_, maximum dose; D_mean_, mean dose; PTV, planning target volume.

### Safety

Treatment-related AEs are detailed in Table 3. Before RT, the most frequent toxicities associated with T-DXd were hematological (overall rate: 22.5%, including 7.5% grade 3 events). One patient developed grade 2 bilateral ILD 1 year before RT and resumed T-DXd therapy after recovery.

**Table 3.** Toxicity description.

Type of adverse event	Toxicity grade	During T-DXt cycle delivered before RT (%)	During T-DXt cycle delivered concurrently with RT (%)	Following T-DXt cycle (%)
**Hematological**				
Neutropenia	Grade 1	0 (0)	2 (5)	0 (0)
	Grade 2	0 (0)	0 (0)	1 (2.5)
	Grade 3	2 (5)	0 (0)	2 (5)
	Grade 4	0 (0)	0 (0)	0 (0)
Anemia	Grade 1	0 (0)	1 (2.5)	0 (0)
	Grade 2	4 (10.0)	1 (2.5)	2 (5)
	Grade 3	3 (7.5)	1 (2.5)	1 (2.5)
	Grade 4	0 (0)	0 (0)	0 (0)
**Non-hematological**				
Esophagitis	Grade 1	0 (0)	0 (0)	2 (5)
	Grade 2	0 (0)	0 (0)	0 (0)
	Grade 3	0 (0)	0 (0)	0 (0)
	Grade 4	0 (0)	0 (0)	0 (0)
Fatigue	Grade 1	2 (5)	2 (5)	1 (2.5)
	Grade ≥2	2 (5)	0 (0)	1 (2.5)
Skin toxicity	Grade 1	0 (0)	0 (0)	0 (0)
	Grade 2	0 (0)	1 (2.5)	4 (10)
	Grade 3	0 (0)	0 (0)	0 (0)
	Grade 4	0 (0)	0 (0)	0 (0)
Diarrhea	Grade 1	0 (0)	1 (2.5)	0 (0)
	Grade 2	0 (0)	0 (0)	0 (0)
	Grade 3	0 (0)	0 (0)	0 (0)
	Grade 4	0 (0)	0 (0)	0 (0)
Increased liver enzymes	Grade 1	0 (0)	2 (5)	2 (5)
	Grade 2	0 (0)	0 (0)	0 (0)
	Grade 3	0 (0)	0 (0)	1 (0)
	Grade 4	0 (0)	0 (0)	0 (0)
ILD	Grade 1	0 (0)	2^a^ (5)	1 (2.5)
	Grade 2	2 (5)	1^a^ (2.5)	0 (0)
	Grade 3	0 (0)	0 (0)	1 (2.5)
	Grade 4	0 (0)	0 (0)	0 (0)

Abbreviations: ILD, interstitial lung disease; T-DXt, Trastuzumab Deruxtecan.

a Radiation-induced pneumonitis (RP).

During RT, 6/40 patients (15.0%) experienced acute toxicity of any grade.

Grade 2-3 toxicity occurred in 3 patients (7.5%). One patient treated for skin metastases developed grade 2 skin toxicity, while another 2 treated to the chest wall experienced grade 2 and grade 3 anemia, respectively; the latter toxicity led to permanent discontinuation of RT. One patient treated with SRT to a paratracheal nodal metastasis developed symptomatic RP 1 month after treatment, which resolved with corticosteroid and antibiotic therapy. Treatment with T-DXd was withheld for one cycle and subsequently resumed.

Overall, 4 out of 40 patients (10%) developed late toxicity: 3 experienced grade 1 toxicity, and 1 experienced grade 3 toxicity. No grade 2 late toxicities were observed. One patient (2.5%) developed grade 3 radionecrosis 16 months after brain SRT, in combination with grade 1 RP. The other 2 patients developed grade 1 RP six months after lung SRT.

The diagnosis of grade 3 radionecrosis was based on sequential brain MRIs with perfusion imaging. After two sequential MRIs suggesting radionecrosis, the patient experienced a rapid clinical decline, with severe neurological symptoms (aphasia) and significant lesion enlargement. The histological confirmation was not possible due to the patient’s refusal of surgery, and the patient died 7 months later due to intracranial leptomeningeal progression.

After RT, 2 additional patients, both treated to pelvic bone metastases, developed ILD (grades 1 and 3, respectively). Treatment details for patients who developed toxicity are summarized in Table 4.

**Table 4.** Per patient toxicity with treatment plan details.

Case	Previous observed toxicity	Acute/Late RT Toxicity (type)	RT technique	RT dose (Total dose/Dose per fraction, Gy)	RT site	PTV volume (cc)	OARs dose (Gy)
Patient1	Neutropenia G3	Late Lung (G1)	SRT	48/12	Right Upper Lobe	6.4	Mean lung right dose = 2.46 Gy
							Lungs V20Gy = 1.84%
		Late Radionecrosis (G1)	SRT	27/9	Frontal lobe tumor bed	23.0	Brain V14 Gy = 58.38 cc
							Brain D20 cc = 28.69 Gy
							Brain V20 Gy = 49.4 cc
Patient 2	None	Acute Diarrhea (G1), Acute Neutropenia (G1)	VMAT	30/3	Sacrum	396	Bowel D_max_ = 34 Gy
							Rectum D_max_ = 28 Gy
Patient 3	Anemia G3	Acute anemia (G3), Neutropenia (G1)	VMAT	54/2	Chest Wall	1169.5	
Patient 4	Fatigue G1 Nausea G1 Liver G1	Acute Anemia (G1) Acute Liver (G1)	SRT	54/18	Left Inferior Lobe	6.7	Liver D_mean_ = 0.1 Gy
							D700 cc = 0.0 Gy
							V10 Gy = 0.0 Gy
Patient 5	None	Late Lung (G1)	SRT	50/10	Liver S8	16.827	Mean lung right dose = 0.377 Gy
							Lungs V20 Gy = 0.0%
		Late Radionecrosis (G3)	SRT	27/9	Left parietal lobe	3.7	Brain V14 Gy = 15.74 cc
							Brain D20 cc = 12.2 Gy
							Brain V20 Gy = 14.4 cc
Patient 6	Anemia G2	Late Lung (G1)	SRT	48/6	Right hilum	19.86	Mean right lung dose = 4.670
Patient 7	None	Acute Liver (G1)	SRT	50/10	IV liver segment	15.93	Liver D_mean_ = 14.9 Gy
							D700 cc = 9.5 Gy
							V10 Gy = 58.2%
Patient 8	None	Acute skin dermatitis (G2)	VMAT	48/1.5	Chest Wall	1343.4	
Patient 9	Anemia G1	Acute anemia (G2)	VMAT	40.05/2.67	Chest Wall	1210.5	
Patient 10	None	Late Radionecrosis (G1)	SRT	27/9	2 parietal left lesions	6.8	Brain V14 Gy = 51.14 cc
							Brain D20 cc = 21.5 Gy
							Brain V20Gy = 24.25 cc
Patient 11	None	Subacute radiation pneumonitis (G2)	SRT	50/5	Right paratracheal node (station 4R)	4.4	Mean lung dose (bilateral) = 0. 679 Gy
							Mean lung dose (right) = 3.569 Gy
							Lungs V20Gy = 0.71%
Patient 12	Fatigue G1	Fatigue G1	SRT	30/6	Left Humeral Head	24.2	

Abbreviations: OARs, organs at risk; SRT, stereotactic body radiotherapy; VMAT, volumetric modulated arc therapy.

## Discussion

T-DXd is playing an increasingly important role in the treatment of HER2-positive and HER2-low metastatic patients. However, despite its undeniable efficacy, data from randomized studies and real-world evidence reveal a significant share of treatment-related AEs, the most common being nausea, neutropenia, and asthenia.^3^^,^^7^^,^^9^^,^^10^ Therefore, particular attention must be paid when combining radiation therapy with this treatment. Currently, insufficient data exist regarding the safety of its combination with RT, leaving a gap in understanding its full potential and associated risks.

To the best of our knowledge, this is the first prospective report analyzing the safety of combining RT and T-DXd in a cohort of patients treated at a single institution. The report provides detailed information on RT protocols and dose exposure to OARs for all administered courses. Overall, in 40 patients treated across 98 sites of disease, the combination was well-tolerated, with side effects observed in 12 out of 40 patients (30.0%) and only one treatment interruption (2.5%) due to grade 3 hematological toxicity.

Interestingly, in a few cases, grade 1 toxicity was observed in patients who received very low mean doses to the affected organ (eg, <3 Gy to the lung or <0.5 Gy to the liver). Although these doses are below the typical thresholds for radiation-­induced damage, a potential causal role of T-DXd or a synergistic effect with T-DXd cannot be excluded.

In the DESTINY-Breast trials,^3^^,^^7^^,^^10^ grade ≥2 hematologic ­toxicities—such as neutropenia, anemia, and thrombocytopenia—were reported in approximately 20%-25%, 14%-20%, and 10%-15% of patients, respectively. Also, grade ≥3 events—such as neutropenia, anemia, nausea, and ILD—were reported in up to 18% of cases, depending on the specific toxicity. While COMBART study lacks a monotherapy control arm, in our cohort, the rate of hematologic toxicity ≥ grade 2 was 10% for neutropenia and 22.5% for anemia, and the incidence of grade ≥3 toxicities was lower than those reported in phase III trials of T-DXd alone, suggesting that the addition of RT did not significantly exacerbate systemic toxicity. Notably, hematologic toxicity remains the most relevant concern, and ILD/pneumonitis requires close monitoring given the known risks with both T-DXd and thoracic RT.

Regarding pulmonary toxicities, the data are encouraging. One patient who developed ILD during prior cycles of T-DXd was able to recover, resume T-DXd, and receive SRT for progressive breast tumor 1 year later without experiencing ILD recurrence. In this series, 34 thoracic lesions (including lung, bones, breast, mediastinal, and supraclavicular nodes lesions) were treated. One patient treated to a right paratracheal node developed symptomatic RP one month after SRT, which resumed after medical therapy. The area of lung consolidation observed on chest CT overlapped with the 10 Gy isodose line, supporting the radiation-induced effect. This patient also had received a previous SRT treatment to a right upper lobe lung metastasis 3 months before this treatment during maintenance therapy with Trastuzumab-Pertuzumab. We believe that the cumulative contribution of the 2 treatments may have determined the toxicity, even if a role of concomitant T-DXd in enhancing radiotherapy-related side effects cannot be excluded.

Two patients treated for lung metastases and one patient for an S8 liver segment lesion experienced low-grade, asymptomatic lung toxicity. The pulmonary alterations observed 6 months post-RT were localized exclusively at the treated site and not widespread. These findings were classified as radiation-induced grade 1 pneumonitis, with T-DXd-related ILD excluded after multidisciplinary review. Consequently, T-DXd was not interrupted for any of these patients.

Three cases of radionecrosis (RN), diagnosed on the basis of sequential perfusion-based MRIs, one symptomatic, were observed among patients treated brain lesions. Dosimetric healthy brain parameters (V14, D20cc, and V20Gy) were exceeded in patients experiencing grade 1 RN but not in the patient presenting with G3 toxicity. This finding, even if the origin of RN is multifactorial, seems consistent with the literature data reporting an increased risk of symptomatic radionecrosis in patients receiving therapy with ADCs. For example, Lebow et al.^11^ reported a 7.1% rate of symptomatic RN among 42 patients treated with ADCs (14 with T-DXd), compared to 0.7% in patients who did not receive concurrent ADCs. Similarly, Koide et al.^12^ analyzed 48 patients treated with ADCs, 19 concurrently, and found a 27% incidence of symptomatic RN in those receiving concurrent RT vs 7% in non-concurrent cases. These findings highlight the importance of addressing factors such as RT dose and treatment volumes in a larger cohort. The diagnosis of radionecrosis remains challenging, as even advanced MRI techniques, such as perfusion imaging, can support but not definitively confirm the diagnosis.

Bouziane J et al.^13^ presented retrospective data from 33 patients who underwent a total of 41 RT treatments, with a median follow-up of approximately 11 months. Their study concluded that the combination of RT and T-DXd is feasible and associated with limited toxicity. Grade 2 toxicity was observed in 21.2% of cases, with no grade 3 toxicities reported. The most common acute grade 1 toxicity was nausea, while grade 2 toxicities included asthenia, mucositis, cardiac decompensation, and diarrhea. Notably, no patients in their study were treated for lung metastases. However, 5 patients discontinued T-DXd due to systemic treatment-related toxicities.

More recently, Debbi et al.^14^ reported the results of the TENDANCE study, which represents the largest published series on concurrent T-DXd and RT in metastatic breast cancer. This retrospective and multicentric study included 54 patients with HER2-positive or HER2-low disease treated with concurrent RT (mainly palliative, 72.2%) and T-DXd therapy. With a median follow-up of 9 months, the main toxicity observed was grade 1-2 asthenia, reported in 51.8% of the study population. Also, 3 patients presented grade 1 ILD, which occurred in the context of breast irradiation and thoracic spine irradiation. Moreover, the incidence of RN was similar to ours, with 3 cases of grade 1 RN reported. No patient discontinued T-DXd related to RT.

In our population, only one patient discontinued RT due to grade 3 anemia, while no patients discontinued T-DXd. Interestingly, patients treated with ablative intent achieved a PFS2 approaching 1 year, suggesting a potential role for SBRT in prolonging the benefits of ongoing systemic therapy.

In conclusion, the safety data for the combination of RT and T-DXd are encouraging. Most non-hematologic toxicities appear to be related to RT, while hematologic toxicities are likely influenced by T-DXd and require close monitoring. Further efforts are needed to better understand, in larger cohorts, the risks of symptomatic radionecrosis in patients with brain metastases undergoing stereotactic RT or a potential influence on the development of RT-related pulmonary toxicity associated with the concomitant administration of T-DXd.

## Data Availability

The data underlying this article will be shared on reasonable request to the corresponding author.
